# Exploring the presence of oral bacteria in non-oral sites of patients with cardiovascular diseases using whole metagenomic data

**DOI:** 10.1038/s41598-023-50891-x

**Published:** 2024-01-17

**Authors:** Aditi Chopra, Ricardo Franco-Duarte, Anjale Rajagopal, Phannaphat Choowong, Pedro Soares, Teresa Rito, Joerg Eberhard, Thilini N. Jayasinghe

**Affiliations:** 1 Department of Periodontology, Manipal College of Dental Sciences, Manipal, Manipal Academy of Higher Education, Manipal, Karnataka India; 2 Department of Biology, CBMA (Center of Molecular and Environmental Biology), University of Minho, Braga, Portugal; 3 Institute of Science and Innovation for Biosustainability (IB-S), University of Minho, Braga, Portugal; 4 School of Dentistry, Faculty of Medicine and Health, The University of Sydney, University of Sydney, Sydney, Australia; 5 The Charles Perkins Centre, The University of Sydney, University of Sydney, Sydney, Australia

**Keywords:** Microbiology, Molecular biology, Cardiology, Molecular medicine

## Abstract

Cardiovascular diseases (CVDs) encompass various conditions affecting the heart and its blood vessels and are often linked with oral microbes. Our data analysis aimed to identify oral bacteria from other non-oral sites (i.e., gut, arterial plaque and cultured blood) that could be linked with CVDs. Taxonomic profiling identified bacteria to the species level and compared with the Human Oral Microbiome Database (HOMD). The oral bacteria in the gut, cultured blood and arterial plaque samples were catalogued, with their average frequency calculated for each sample. Additionally, data were filtered by comparison with the Human Microbiome Project (HMP) database. We identified 17,243 microbial species, of which 410 were present in the HOMD database and further denominated as “oral”, and were found in at least one gut sample, but only 221 and 169 species were identified in the cultured blood and plaque samples, respectively. Of the 410 species, 153 were present solely in oral-associated environments after comparison with the HMP database, irrespective of their presence in other body sites. Our results suggest a potential connection between the presence of specific species of oral bacterial and occurrence of CVDs. Detecting these oral bacterial species in non-oral sites of patients with CVDs could help uncover the link between oral health and general health, including cardiovascular conditions via bacterial translocation.

## Introduction

Cardiovascular diseases (CVDs) encompass various conditions affecting the heart and its blood vessels. Within the group of CVDs, the following diseases are often included: (a) atherosclerotic cardiovascular disease (coronary, cerebrovascular, and peripheral vascular disease), (b) valvular heart disease, (c) heart failure and cardiomyopathies, (d) arrhythmias, (e) infective and autoimmune conditions (including infective endocarditis), and (f) hypertension^1^. CVDs are one of the most common causes of death worldwide^2^, leading to approximately 19.05 million deaths in 2020^3^. Since the global burden of CVDs is rapidly increasing, it is crucial to identify its risk factors for effective prevention. Ethnicity^4^, age^5^, family history^6^, dyslipidemia^7^, hypertension^8^, tobacco smoke^9^, excess body weight^10^, physical inactivity and sedentary lifestyle^11^, and diabetes mellitus^12,13^ are some of the common risk factors for CVDs^13,14^. Recent studies have demonstrated the link between infection and inflammation in atherosclerosis^15^. In that scenario, the role of oral dysbiosis in systemic inflammation, immune cross-reactivity and its association with CVDs is gaining attention^16,17^.

The oral cavity and the gut, which are at either end of the gastrointestinal tract (GIT), harbor thousands of microbes. The gut is the largest and most well-characterised microbial ecosystem in the human body, with a microbial ecosystem of approximately 500 to 1000 species from more than 50 different phyla^18^. The major anaerobic microorganisms of the gut belong to four major phyla: Bacteroidetes, Firmicutes, Proteobacteria, and Verrucomicrobia. Phyla Bacteroidetes and Firmicutes account for more than 90% of the total microbiota^19,20^. Studies in mice and humans have shown evidence that the translocation of bacteria from the oral cavity can alter the gut microbiome^21,22^, which contains approximately 619 different bacterial taxa from 13 different phyla of bacteria^23,24^.

Oral bacteria can migrate into the gut through hematogenous and enteral routes^25,26^. The hematogenous route is via the blood vessels (systemic circulation) around the gingival and periodontal tissues and the enteral route is the direct entry of bacteria into the gut via swallowed saliva. The entry of bacteria into the systemic circulation (bacteraemia) increases systemic inflammation and leads to various organ dysfunction. Oral bacteria invasion into circulation has been linked with various systemic diseases such as diabetes, rheumatoid arthritis, non-alcoholic fatty liver disease, inflammatory bowel disease, pancreatic cancer, colorectal cancer, and so on^27–29^. The oral bacteria can even influence the microbiome at other body sites and result in microbial dysbiosis^22^. Oral bacteria and their role in gut dysbiosis is considered as potential cause of many systemic diseases. Humans swallow approximately 1.5 L of saliva per day containing numerous bacteria^30,31^ and yeasts^32^. Although gastric acidity is a barrier for many ingested oral bacteria, some bacteria such as *Porphyromonas gingivalis* can tolerate the harsh acidic environment and cross the gut barrier to reach the systemic circulation and other distant organs’ barrier^33^. Oral bacteria have been identified in kidneys, uterine tissues, liver and intestine. Many oral bacteria such as *Prevotella intermedia, P. gingivalis*, *Fusobacterium nucleatum* and *Helicobacter pylori* have been identified in the colonies of arterial plaques, pancreatic tissues, and gut mucosal tissues^34–38^.

**Figure 1:**
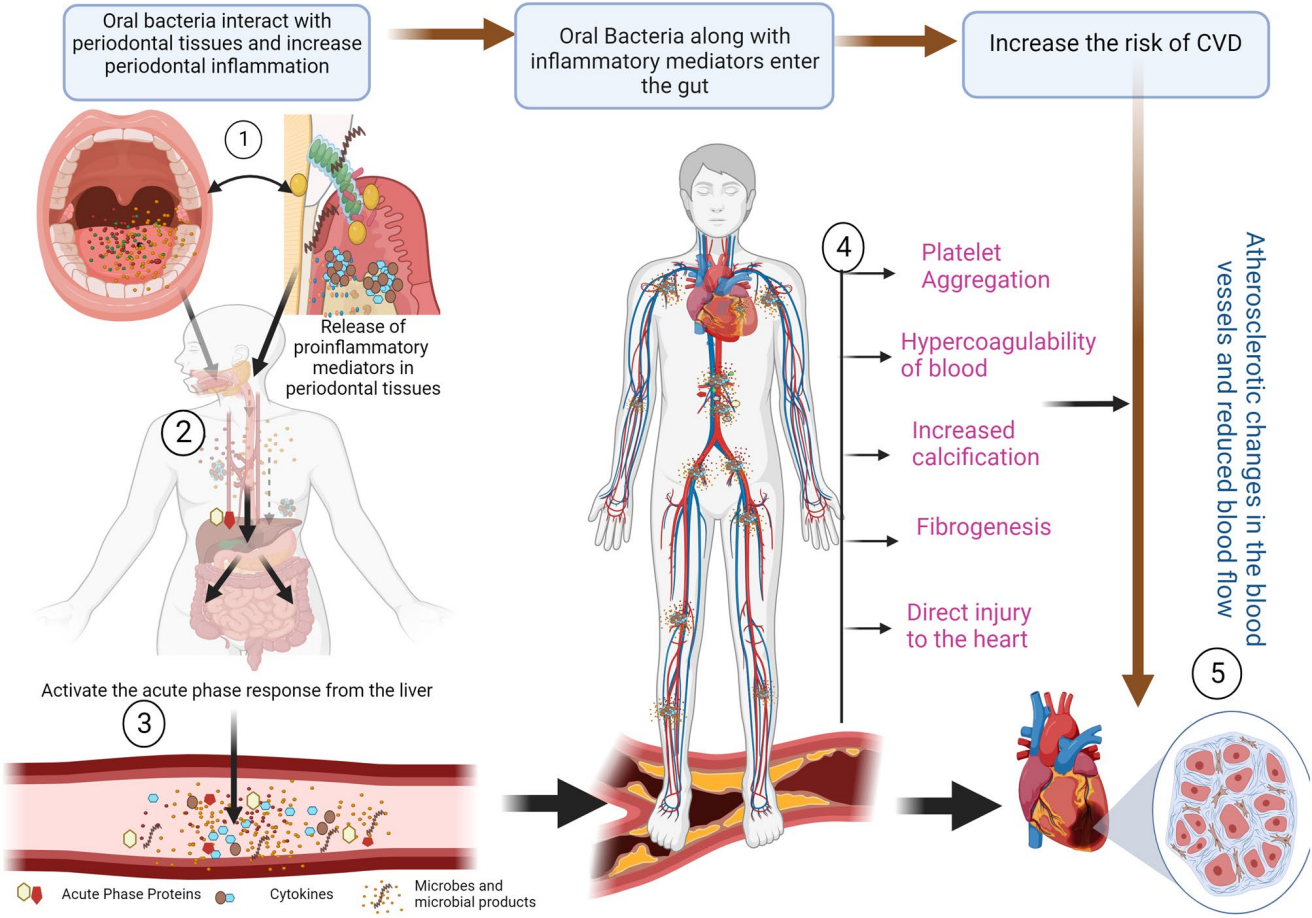
Schematic representation of how oral bacteria can increase the risk of cardiovascular diseases (CVDs): (1) The oral cavity contains millions of microorganisms, which comprise bacteria, viruses, fungi, and protozoa which interact with the gingival and periodontal tissues and lead to periodontal inflammation. (2) This host microbial interaction triggers an inflammatory response with massive release of proinflammatory mediators such as tumour necrosis factors (TNF), interleukins (IL-1, IL6, IL8), free radicals or reactive oxygen species (ROS). These proinflammatory mediators along with microbial by-products reach the systemic circulation either directly via swallowing or the blood vessels surrounding the teeth. (3) The microbes along with inflammatory mediators increase systemic inflammation and activates the release of various Acute Phase Proteins such as C-reactive protein (CRP), pentraxin, and fibrinogen from the liver. (4) These Acute Phase proteins, particularly fibrinogen and CRP increase the viscosity of the blood, injure the endothelium of the blood vessels and induce platelet aggregation, and alter lipid metabolism. This increases the risk of atherosclerosis and thrombus formation in the blood vessels. (5) Thrombus formation constricts the blood vessel and reduces the blood supply to the heart and organs [Created with BioRender.com].

The invasion of oral bacteria from the oral cavity into systemic circulation activates a series of immuno-inflammatory response that cause a massive release of pro-inflammatory mediators (IL-1, IL-6, TNF-α, and MCP-1), acute phase proteins, C-reactive protein (CRP), fibrinogen, and free radicals into the systemic circulation^39^. These chemical mediators of inflammation affect the cardiovascular system as they alter lipid metabolism, causes endothelial injury and increase the hypercoagulability of the blood and platelets. This, in turn, increases the risk of atherosclerosis, arterial calcification, fibrogenesis, and cardiovascular tissue injury^40–43^ (Fig. 1). Studies have confirmed the link between increased oral bacteria, bacteraemia and CVDs^44–52^. Among all the oral microbes, bacteria associated with periodontal disease, particularly those belonging to the red and orange complex have been linked with system diseases such as CVDs^53,54^. Jafarzadeh et al*.*^55^ also found that the causal odds of facing CVDs are increased by 1.52-fold in patients with prior instances of bacteraemia. The odds of having CVDs due to sepsis are increased by 2.39-fold^55^. While the role of the oral microbiome and its influence on the development of CVDs is gaining increased consensus, the potential for oral microbial translocation to non-oral sites leading to systemic inflammation in patients with CVDs is a noteworthy concern^56–58^.

Only a limited number of studies have analysed the whole genomic sequencing data of the gut, arterial plaque or blood microbiome, especially of patients with CVDs^59–61^. None of these studies simultaneously reported bacteria in the gut, oral cavity, blood or arterial plaque samples. Since increasing evidence has suggested that oral microbes can spread throughout the body and have been found in various systemic disease conditions^62,63^, we carried out an analysis of whole metagenomic data to identify the oral bacterial species in different biological samples of subjects with various CVDs. In this study, we examined publicly available whole metagenomic data from six distinct studies, encompassing samples obtained from the gut, cultured blood, and arterial plaque. Our main aim was to identify the oral bacteria in gut, cultured blood, and arterial plaque samples of patients with CVDs. This review will help strengthen the potential association between various oral bacterial species and the incidence of CVDs.

## Methods

### Search strategy, information sources, and keywords

MeSH terms or free texts for this secondary data analysis were defined in three main areas of interest: whole-metagenomic data, bacteria, and cardiovascular diseases. To maximise the search inclusiveness, all relevant/synonym search terms of these three keywords were examined (Supplementary Table S1). Four scientific and electronic databases were searched (PubMed, Ovid Medline, EMBASE, and Web of Science) in August 2022. Articles written in English from the last 22 years (2000 to 2022) were considered.

### Inclusion and exclusion criteria

All studies reporting the whole-metagenomic microbial data of adult populations with the following CVDs were included: atherosclerotic cardiovascular disease including stroke, valvular heart disease, heart failure, cardiomyopathy, arrhythmias, infective and autoimmune conditions (including infective endocarditis), and hypertension. Studies reporting individual bacteria isolated from samples, such as strain typing and evolution tracking, were excluded. Additionally, studies that reported bacteria from oral samples such as gingival tissues, plaque, gingival crevicular fluid, oral swabs, or salivary samples alone were also excluded. Studies in which English translation for full text was not available were excluded. All systematic reviews, scoping reviews, narrative reviews, book reviews, commentaries, perspectives, case reports, case series, and editor letters were excluded.

### Data extraction

The bibliographic search results were transferred into the Mendeley reference manager (version 2.66.0), thereby removing duplicates. An initial set of 2995 articles were considered. Two reviewers (A.C. and A.R.) independently performed the searches and screened the articles for title and abstract based on the eligibility criteria. Any disagreements were mutually discussed between the two researchers, and a final decision was made after mutual discussion with two additional reviewers (T.J. and P.C.). Full-text screening was performed independently by four authors (A.C.; A.R.; T.J.; P.C.). All disagreements were discussed among the authors, and after mutual consensus, the final set of 6 articles was selected for data extraction. The following metadata were extracted from each bibliographic study:

1. *Methods* type of study design, country of the study.
2. *Participants* number of participants in each group recruited, number of participants analysed, mean age (in years), and type of CVD.
3. The type of taxonomic analysis used for whole-genomic or meta-genomic sequencing and comparison to the HOMD and HMP databases to confirm that bacteria have an oral presence.

### Metagenomic data analysis

Raw metagenomic data from patients with CVDs (originally sequenced using paired-end Illumina technology) in FASTQ format from selected studies were extracted. A total of 458 samples were considered, from the six selected studies. All the BioSample accession numbers including SRR and ERR numbers of the data files used in this analysis have been stated in Supplementary Table S2. Taxonomic profiling of the entire metagenomic data sets was performed with Kaiju software v.1.9.0^64^ using the *nr_euk* database, available in the kaiju web-server (71 GB; 2022-03-10), and standard parameters as described in Santos-Pereira et al*.*^65^. The Kaiju2table script was used to convert Kaiju’s output files into a summary table for taxonomic ranks.

Bacteria identified to the species level and detected via the Human Oral Microbiome Database (HOMD; https://www.homd.org/) were classified as “oral bacteria”. Furthermore, data were filtered after comparison with the Human Microbiome Project (HMP; https://hmpdacc.org/) to identify those commonly found in oral environments, irrespective of their mutual presence in other body matrices. In particular, we downloaded the entire database considering species abundance in each of the 15 body site locations detailed (Supplementary Table S3). Data were collected and analysed in R studio (v.2021.9.2.0) to calculate the frequency of each microbial species in each study, including their overall frequency across the seven analysed studies. In detail, following the enumeration of all bacterial species found in at least one sample, we collected a range of data for each bacterial species. This data included the overall frequency, which considered the presence of each species across the total of 458 samples, the number of files analysed, the total number of files, the average frequency of the bacteria within each given sample, the sum of the frequency of each species in each particular study, and whether the species were present or absent in the HOMD database (Supplementary Table S3). After identification of the microbial species present in each sample for each study, we calculated their frequency within each study, and employed Relative Species Abundance (RSA) as a normalisation method, being used as a measure of how common a species is relative to other species in a defined sample and study. The taxonomy of each bacterial species (Domain; Phylum; Class; Order; Family; Genus; Species) was identified along with location at other body sites, cultivability, phenotypic characteristics, and presence in different oral niches (according to the HMP dataset). The data for each bacterial species related to disease, especially to CVDs, were identified via Medline on the PubMed platform, NCBI—Genome, and the Bacterial Diversity Meta-database (BacDive). These bacteria were also compared to healthy samples from the (HMP) to find oral microorganisms linked to CVDs.

## Results

### Characteristics of the samples considered

A total of 2995 titles and abstracts were considered. After removing duplicates, 2587 research articles were retrieved. After screening for inclusion and exclusion criteria, a total of 101 studies were included for full-text screening. After an additional assessment, 20 studies were included. However, six studies were considered for the final analysis due to the unavailability of original sequencing data from 14 studies. The characteristics of each included study are given in Table 1. The considered studies contain data obtained from different geographical origins—Italy^60^, France^66^, Singapore^61^, Sweden^59^, and China^67,68^—and from different types of CVDs—atherosclerosis^60,68^, atrial fibrillation^67^, ischemic attacks^61^, and infective endocarditis^66^.

**Table 1:** Detailed characteristics of the studies included.

References	Jie et al.^68^	Zuo et al.^67^	Karlsson et al.^59^	Baragetti et al.^60^	Mitra et al.^61^	Million et al.^66^
Origin	Gut	Gut	Gut	Gut	Plaque	Blood culture
Type of CVD	Atherosclerosis	Atrial fibrillation	Atherosclerosis	Atherosclerosis	Ischemic attacks	Infective endocarditis
Country	China	China	Sweden	Italy	Singapore	France
Number of participants (age range)	405 (61 years)	100 (57–71 years)	410 (65 ± 5 years)	345 (67 ± 11 years)	22 (not mentioned)	10 (not mentioned)
Number of samples analysed	214	44	26	165	7	4
Average percentage of reads identified to species-level (min–max)	88.0 (21–97)	93.3 (86–96)	77.30 (53–87)	95.18 (63–100)	13.28 (13–15)	39 (16–84)
Average percentage of bacteria identified to species-level (min–max)	87.43 (68–96)	93.09 (86–97)	76.15 (53–86)	80.12 (18–96)	11 (10–12)	34.75 (13–83)
Average number of reads per sample	2.6 × 10^7^	2.5 × 10^7^	3.0 × 10^7^	1.3 × 10^7^	3.3 × 10^6^	1.3 × 10^7^
Three most abundant bacterial species	*Escherichia coli, Klebsiella pneumoniae, Bifidobacterium longum*	*Escherichia coli, Bifidobacterium longum, Klebsiella pneumoniae*	*Escherichia coli, Staphylococcus haemolyticus, Pseudomonas fluorescens*	*Escherichia coli, Bifidobacterium longum, Klebsiella pneumoniae*	*Acinetobacter baumannii, Mycobacterium tuberculosis, Enterococcus faecalis*	*Moraxella osloensis, Acinetobacter baumannii, Enhydrobacter aerosaccus*
Most abundant oral bacterial species	*Streptococcus salivarius*	*Streptococcus salivarius*	*Neisseria meningitidis*	*Streptococcus salivarius*	*Lactobacillus crispatus*	*Cutibacterium acnes*

A total of 458 samples were considered in our analysis, obtained from individuals with a certain CVD condition. From these, 447 samples were obtained from the gut, four were from cultured blood, and seven were from arterial plaque samples. Detailed characteristics (type of CVD, average age or age range of the participants, number of samples analysed, total species identified) for each research study considered are described in Table 1.

### Microbes identified in patients with CVDs

A total of 17,243 microbial species (bacteria and fungi) were identified at the species level. Considering the software and database employed, the average percentage of metagenomic reads identified up to the species level ranged from 10% (in atherosclerotic plaque samples) to 97% (in the gut). From the 17,243 microbes identified, 410 bacterial species were found to be present in the HOMD database (Supplementary Table S4), which we further denominated as “oral”, and from these, we filtered the ones commonly found in oral environments as identified in the Human Microbiome Project (HMP), irrespective of their mutual presence in other body matrices (Supplementary Table S5).

**Figure 2:**
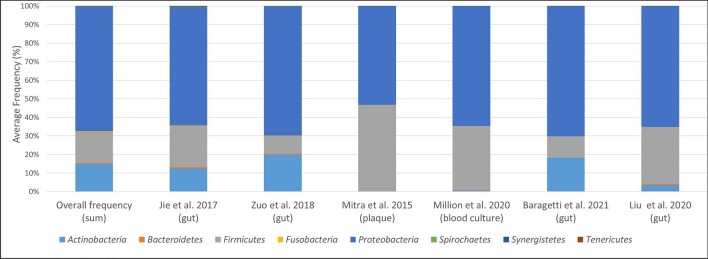
Bacterial phyla identified in each study.

**Figure 3:**
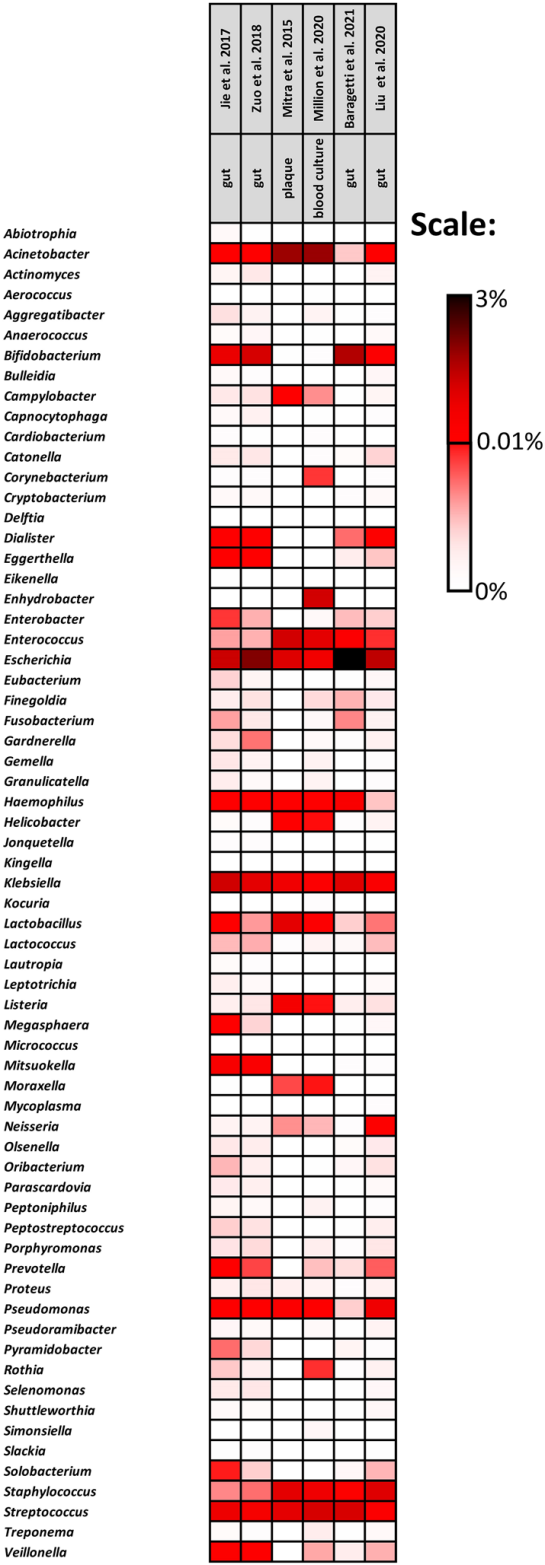
Heatmap representing the abundance (relative frequency) of each bacterial genera identified per study.

The above-identified 410 “oral” bacterial species were under eight phyla: Actinobacteria, Bacteroidetes, Firmicutes, Fusobacterium, Proteobacteria, Spirochetes, Synergistetes and Tenericutes (Fig. 2). Heatmap in Fig. 3 illustrates the distribution of bacteria in each study at the genus level, together with their abundance as calculated within each study.

#### Oral bacteria present in the gut of patients with CVDs

*Escherichia, Klebsiella, Streptococcus* and *Bifidobacterium* were identified as the most representative genera (Fig. 3) in the faecal samples of patients with CVDs. In particular, *Pseudomonas* bacteria were highly abundant in the samples from Karlsson et al*.*^59^, while the genera *Bifidobacterium* and *Streptococcus* had a lower abundance in comparison with the other studies. Regarding bacterial phyla (Fig. 2), Karlsson et al*.*^59^ also showed a reduction in the overall frequency of Actinobacteria and an increase in Firmicutes phyla, a scenario more similar to that obtained in samples from plaques and cultured blood.

**Figure 4:**
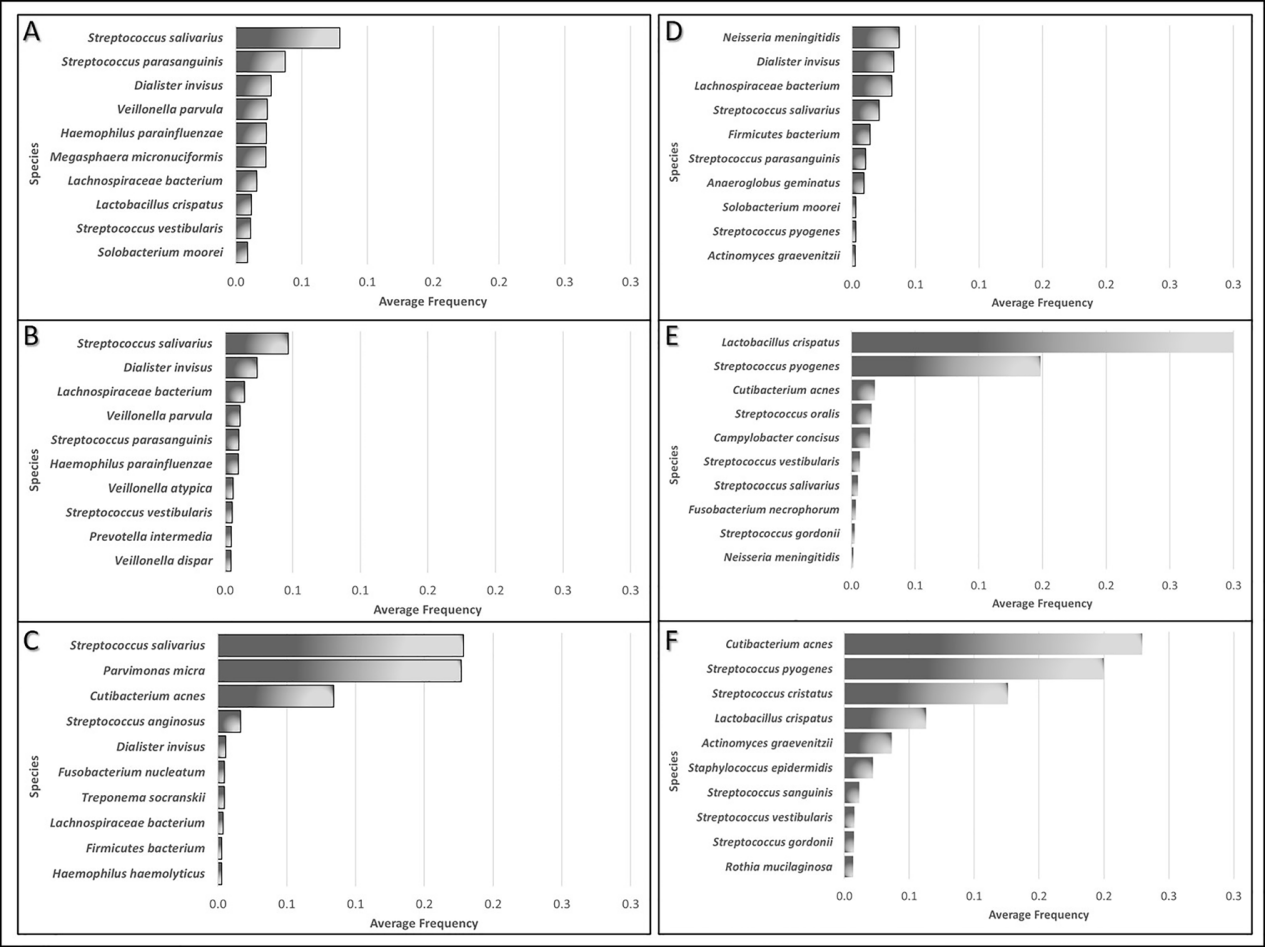
Ten most abundant bacterial species identified in samples from the following studies: (**A**) Jie et al*.*^68^; (**B**) Zuo et al*.*^67^; (**C**) Baragetti et al*.*^60^; (**D**) Karlsson et al*.*^59^; (**E**) Mitra et al.^61^; (**F**) Million et al.^66^. The results of *Escherichia coli* were omitted for simplification since this species was detected in all samples from all studies.

When detailing the 410 oral bacterial species, *Escherichia coli* was found to be the most common bacteria in the gut, as expected, with an overall frequency of 1.0 across all the analysed samples, which indicates its presence in all the samples from all the studies. Figure 4 details the ten most abundant bacterial species found in gut samples of four studies after the omission of *E. coli* results, for simplification, revealing *Streptococcus salivarius* as the most abundant species in three of the four studies analysing gut samples (Fig. 4A–C). In contrast, *Neisseria meningitidis* peaked in the remaining study (Fig. 4). Only three bacterial species (*Streptococcus salivarius*, *Dialister invisus* and *Lachnospiraceae bacterium*) were among the ten most representative bacteria in all four studies.

#### Oral bacteria in the cultured blood and plaque samples of patients with CVDs

Contrary to the scenario observed for the faeces samples, in samples obtained from cultured blood and plaque samples, the most frequent genera were *Acinetobacter* and *Enterococcus*, which were almost absent in gut samples. When comparing cultured blood and plaque samples, some distinct differences can be highlighted in terms of the most represented genera: (1) cultured blood samples showed a high frequency of *Enhydrobacter* bacteria (average frequency of 0.5), which were almost absent in plaque samples; (2) *Streptococcus* bacteria showed a higher frequency in cultured blood samples among all datasets analysed; and (3) *Lactobacillus* was detected in plaque samples at average frequencies above 0.3, being only residually detected in samples from all other origins.

When considering identifications at the species level, all 410 different oral bacterial species were found in at least one gut sample, but only 221 and 169 species were identified in the cultured blood and plaque samples, respectively, corresponding to 54% and 41% of the species identified in gut samples, respectively. Within the bacteria identified, six species were commonly categorised among the ten most represented bacterial species, both in plaque samples and blood cultures (Fig. 4E,F, respectively)—*Lactobacillus crispatus, Cutibacterium acnes, Streptococcus gordonii, Streptococcus pyogenes, Streptococcus salivarius* and *Streptococcus vestibularis*. *Lactobacillus crispatus* was detected at the highest frequency in the plaque samples (average frequency of 0.31). In contrast, *Cutibacterium acnes* was the species peaking in the blood culture samples (average frequency of 0.23). When comparing the most abundant bacteria to the top ten species identified in the gut samples, only *Cutibacterium acnes* bacteria were detected in faeces samples in one study (Fig. 4C) at much lower frequencies, highlighting the differences that the samples´ origins cause.

### Oral bacteria linked with oral health, periodontal disease and caries and their prevalence in the included studies

Our analysis detected 36 bacterial species that are known to be related to oral health (Table 2), as identified via Medline on the PubMed platform, NCBI—Genome, and the Bacterial Diversity Meta-database (BacDive). *Haemophilus parainfluenza* was detected in all studies and identified at the highest frequency (overall average frequency of 1.4 × 10^–2^). Other genera commonly associated with oral health identified in our analysis included *Corynebacterium*, *Gemella*, *Haemophilus*, *Kingella*, *Neisseria* and *Rothia* (Table 2).

**Table 2:** Oral bacterial species and their average frequency quantified using our data analysis pipeline as being linked with oral health, periodontal disease, and caries.

Bacteria related to oral health		
*Capnocytophaga sputigena*—1.75 × 10^–4^ *Capnocytophaga ochracea*—2.9 × 10^–5^ *Cardiobacterium hominis*—6.1 × 10^–5^ *Corynebacterium durum*—8.2 × 10^–4^ *Corynebacterium matruchotii*—4.1 × 10^–5^ *Corynebacterium mucifaciens*—1.0 × 10^–8^ *Gemella bergeri*—1 × 10^–5^ *Gemella haemolysans*—5.8 × 10^–4^ *Gemella morbillorum*—8.4 × 10^–4^ *Gemella sanguinis*—3.9 × 10^–3^ *Granulicatella adiacens*—3.0 × 10^–4^ *Haemophilus parainfluenzae*—1.4 × 10^–2^	*Haemophilus aegyptius*—1.0 × 10^–6^ *Kingella denitrificans*—3.0 × 10^–6^ *Kingella oralis*—9.0 × 10^–7^ *Mycoplasma salivarium*—7.0 × 10^–7^ *Neisseria cinerea*—3.0 × 10^–6^ *Neisseria elongata*—5.0 × 10^–5^ *Neisseria flava*—7.0 × 10^–9^ *Neisseria flavescens*—2.0 × 10^–5^ *Neisseria lactamica*—2.0 × 10^–6^ *Neisseria meningitidis*—1.2 × 10^–3^ *Neisseria weaveri*—8.0 × 10^–8^ *Neisseria mucosa*—4.0 × 10^–6^	*Neisseria oralis*—2.0 × 10^–8^ *Neisseria sicca*—3.0 × 10^–5^ *Neisseria subflava*—4.9 × 10^–6^ *Prevotella oris*—2.8 × 10^–4^ *Peptoniphilus lacrimalis*—1.45 × 10^–4^ *Porphyromonas catoniae*—4.3 × 10^–5^ *Rothia aeria*—1.8 × 10^–4^ *Rothia dentocariosa*—7.0 × 10^–5^ *Rothia mucilaginosa*—1.2 × 10^–3^ *Selenomonas noxia*—1.4 × 10^–4^ *Selenomonas artemidis*—4.9 × 10^–5^ *Streptococcus sanguinis*—4.46 × 10^–3^

Bacteria related to periodontal disease according to different microbial complex		
Red complex	Orange complex	Green complex
*Porphyromonas gingivalis*—5.3 × 10^–4^ *Treponema denticola*—7.0 × 10^–5^ *Tannerella forsythia*—6.6 × 10^–4^	*Prevotella intermedia*—2.8 × 10^–3^ *Prevotella nigrescens*—4.0 × 10^–4^ *Fusobacterium nucleatum*—3.12 × 10^–3^ *Streptococcus constellatus*—4.3 × 10^–4^ *Campylobacter showae*—6.0 × 10^–5^ *Campylobacter gracilis*—1.2 × 10^–4^ *Campylobacter rectus*—1.0 × 10^–5^ *Campylobacter concisus*—5.8 × 10^–4^	*Eikenella corrodens*—3.0 × 10^–3^ *Capnocytophaga gingivalis*—2.0 × 10^–5^ *Capnocytophaga sputigena*—1.7 × 10^–4^ *Capnocytophaga ochracea*—2.9 × 10^–5^ *Campylobacter concisus*—5.8 × 10^–4^ *Aggregatibacter actinomycetemcomitans*—1.0 × 10^–4^

Yellow complex	Purple complex	Blue complex
*Streptococcus sanguinis*—4.5 × 10^–3^ *Streptococcus oralis*—4.0 × 10^–3^ *Streptococcus mitis*—3.5 × 10^–3^ *Streptococcus gordonii*—2.54 × 10^–3^ *Streptococcus intermedius*—8.4 × 10^–4^ *Streptococcus anginosus*—6.36 × 10^–3^	*Veillonella parvula*—1.5 × 10^–2^	*Actinomyces naeslundii*—3.9 × 10^–4^ *Actinomyces viscosus*—2.0 × 10^–5^
Novel periodontal pathogens		
*Acinetobacter baumannii*—2.8 × 10^–2^ *Anaeroglobus geminatus*—1.63 × 10^–3^ *Bulleidia extructa*—1.6 × 10^–4^ *Comamonas testosteroni*—2.0 × 10^–5^ *Dialister invisus*—1.86 × 10^–2^ *[Eubacterium] sulci*—1.24 × 10^–3^ *[Eubacterium] nodatum*—2.5 × 10^–4^ *Filifactor alocis*—9.0 × 10^–5^ *Fretibacterium fastidiosum*—1.5 × 10^–4^ *Fretibacterium fastidiosum*—1.5 × 10^–4^	*Johnsonella ignava*—2.9 × 10^–4^ *Leptotrichia buccalis*—1.0 × 10^–5^ *Leptotrichia hofstadii*—4.2 × 10^–4^ *Leptotrichia hongkongensis*—2.0 × 10^–5^ *Mogibacterium diversum*—2.1 × 10^–3^ *Mogibacterium timidum*—4.1 × 10^–3^ *Neisseria lactamica*—2.0 × 10^–5^ *Neisseria elongata*—5.0 × 10^–5^ *Neisseria flavescens*—2.0 × 10^–5^ *Parvimonas micra*—3.6 × 10^–2^	*Pyramidobacter piscolens*—3.36 × 10^–3^ *Peptostreptococcus anaerobius*—2.4 × 10^–4^ *Peptostreptococcus stomatis*—9.4 × 10^–4^ *Solobacterium moorei*—5.13 × 10^–3^ *Scardovia wiggsiae*—7.8 × 10^–4^ *Streptococcus parasanguinis*—2.0 × 10^–2^ *Treponema socranskii*—9.6 × 10^–4^ *Treponema maltophilum*—5.0 × 10^–5^ *Treponema vincentii*—6.0 × 10^–5^

According to the PubMed platform, NCBI—Genome, and the Bacterial Diversity Meta-database (BacDive), out of a total of 410 bacterial species identified in our samples of CVDs patients, twenty-nine periodontal bacterial species were noted to be linked with periodontal and odontogenic diseases. Whitin this list, *Dialister invisus, Parvimonas micra* and *Streptococcus parasanguinis* had the highest overall average frequency. According to the Socransky classification of microbial complex of subgingival periodontal pathogens belonging to the red, orange, green, yellow, purple and blue complexes were detected in the analysed samples from patients with CVDs (Table 2). *P. gingivalis*, a keystone pathogen in the oral cavity and primary cause of periodontal disease, was identified in the faecal samples (average frequency of 5.3 × 10^–4^) and cultured blood samples (average frequency of 3.0 × 10^–4^) of CVD patients. At the same time, it was absent in arterial plaque samples. Among the analysed microbial complexes, the highest number of matched bacterial species identified belonged to the orange complex (8 species). In contrast, only one species from the purple, and two from the blue complexes were identified. From the list of the identified bacteria that are related to the periodontal disease, *Veillonella parvula* (purple complex) was detected at the highest frequencies, followed by *Streptococcus sanguinis* and *Streptococcus oralis* (yellow complex).

### Comparison between identified “oral” bacteria from patients with CVDs to healthy individuals’ microbiome data in HMP

To understand which of the “oral” species shown to be present in CVDs samples were also present in healthy individuals, we compared the 410 species identified in the HOMD database against the list of bacteria catalogued in the HMP database as being present in niches of healthy individuals. In particular, the following niches were evaluated from the HMP database: anterior nares, keratinized gingiva, buccal mucosa, left and right retroauricular crease, palatine tonsils, saliva, stool, subgingival and supragingival plaques, throat, tongue dorsum, vaginal introitus, mid vagina, and posterior fornix. A total of 153 species, out of the 410 oral species, were found in at least one of these environments in healthy subjects (Supplementary Table S3). The tongue dorsum was the location with the highest number of “oral” species catalogued (112), followed by supragingival plaque and buccal mucosa with 101 species each. Eighty-seven bacterial species were catalogued in the subgingival plague samples of healthy HMP subjects. Of these bacteria, *Corynebacterium matruchotii, Haemophilus parainfluenza* and *Rothia dentocariosa* were present in higher abundance in healthy individuals (HMP database), even though the percentages identified in CVDs samples were very small. Similarly, out of 410 bacterial “oral” species identified, 86 bacterial species in the CVDs dataset were present in the saliva of the HMP database. Of these 86 species, *Streptococcus mitis* and *Hemophilus parainfluenza* were the most common in healthy subjects, which is in accordance with the high representativity of these species in CVD samples (Supplementary Table S3).

### Microbial profiles between diseased and controls in the analysed studies

Originally, the majority of the included studies focused on comparing the microbiome profile between participants with CVDs and their respective healthy controls. However, as we identified variations in the microbial profiles, we chose to delve deeper into these comparisons. Specifically, we examined the most abundant microbes (genus/species) present in each study, as summarised in Table 1 and in Supplementary Tables S6 and S7. We then examined original studies to compare these microbes between individuals with CVDs and healthy controls. We identified six genera that were consistently present in both CVDs and control samples: *Escherichia*, *Prevotella*, *Bacteroidetes*, *Eubacterium*, *Lactobacillus*, and *Streptococcus*. Upon examining the data from individual studies (Supplementary Tables S6, S7), we observed distinct variations in the microbial profiles:

i. In the study by Jie et al*.*^68^, a significantly higher abundance of *Bacteroides fragilis*, *Streptococcus salivarius*, *Clostridium nexile*, *Ruminococcus gnavus*, *Ruminococcus torques*, *E. coli*, *Klebsiella pneumoniae*, and *Akkermansia muciniphila* was found in patients with CVDs. Conversely, *Faecalibacterium prausnitzii*, *Prevotella copri*, *Bacteroides uniformis, Bacteroides caccae, Bacteroides dorei, Bacteroides fecis*, *Bacteroides finegoldii* were more abundant in control samples. Furthermore, *Bacteroides xylanisolvens* were more prevalent in controls, while *Bifidobacterium adolescentis* and *Collinsella aerofaciens* exhibited relatively higher abundance in patients compared to controls (p < 0.05 but q > 0.05). Several other species that were more abundant in atherosclerotic patients include *Streptococcus salivarius*, *Streptococcus parasanguinis*, *Streptococcus anginosus*, *Streptococcus vestibularis*, *Streptococcus infantis*, *Streptococcus mitis*, *Streptococcus oralis* and *Streptococcus pneumoniae*. However, butyrate-producing bacteria *Roseburia intestinalis* and *Faecalibacterium prausnitzii* were relatively depleted in the atherosclerotic CVD samples (q < 0.05). Additionally, oral bacteria such as *Streptococcus spp*., *Lactobacillus salivarius*, *Solobacterium moorei*, and *Atopobium parvulum*, were more prevalent in patients with atherosclerosis compared to healthy controls (q < 0.05).
ii. Zuo et al*.*^67^ also noted an increased richness and diversity in the gut microbial profile in patients with atrial fibrillation. Notably, two dominant Enterotypes, *Bacteroides* and *Prevotella*, were identified. In the control group, an equal distribution of samples within both enterotypes was observed (50% of *Bacteroides*, 50% of *Prevotella*). In contrast, patients with atrial fibrillation exhibited a higher percentage of *Bacteroides* (82%) and a lower percentage of *Prevotella* (p = 0.001). The study further highlighted significant differences in genera such as *Blautia*, *Coprobacillus*, *Dorea*, *Enterococcus*, *Streptococcus*, and *Veillonella*) among patients with atrial fibrillation.
iii. Similar findings were reported in the study by Karlsson et al*.*^59^ where the genus *Collinsella* exhibited enrichment in patients with atherosclerosis, while *Eubacterium*, *Roseburia* and certain species of *Bacteroides* were enriched in healthy controls (adjusted p < 0.05). The levels of *Bacteroides*, *Prevotella*, and *Ruminococcus* were also different in patients with CVDs compared to control. Notable variations were observed in the levels of *Bacteroides*, *Prevotella*, and *Ruminococcus* between patients with CVDs and controls. Specifically, *Bacteroides* were less prevalent (p = 0.0048), and *Ruminococcus* were more abundant (p = 0.047) in patients with atherosclerosis compared to controls. Six species (*Bacteroides xylanisolvens, Odoribacter splanchnicus, E. eligens, Roseburia intestinalis, Roseburia inulinivorans*, and *Ruminococcus albus*) were shared between the studies by Jie et al*.*^68^ and Karlsson et al*.*^59^ and these species were found to be more abundant in the control samples. Among these species, Karlsson et al*.*^59^ revealed significantly higher mean abundances of *B. xylanisolvens* and *E. eligens* in the control samples (p < 0.001, q < 0.05). Additionally, two species belonging to the *Roseburia* genus (*R. inulinivorans* and *R. intestinalis*) exhibited enrichment in the control group across both studies, suggesting a potential beneficial role in preventing atherosclerotic diseases.
iv. Furthermore, the comparative analysis of microbial profiles was extended to patients with subclinical carotid atherosclerosis (SCA) and those with carotid Intima-Media Thickness (IMT) compared to individuals not exhibiting these profiles^60^. Notably, subjects with SCA exhibited an elevated relative abundance of members from the *Escherichia* genus (2.8% vs. 1.4%, p = 0.008 in SCA and non-SCA subjects, respectively) and the *Oscillospira* genus (6.5% vs. 5.7%, p = 0.013 in SCA and non-SCA subjects, respectively). The most prevalent family was Ruminococcaceae (32.9%), followed by Lachnospiraceae (19.1%), Bacteroidaceae (15.6%), Veillonellaceae (4.3%), and Prevotellaceae (4.1%). Noteworthy genera included *Faecalibacterium* (15.9% on average), *Bacteroides* (15.6%), *Oscillospira* (5.8%), *Roseburia* (4.7%), *Prevotella* (4.0%), *Ruminococcus* (3.8%), as well as unidentified members of the Ruminococcaceae (6.4%) and Lachnospiraceae families (5.0%). Metagenomic comparisons between subjects without IMT/SCA and those with IMT/SCA unveiled an increase in the abundance of *E. coli*, as well as members of the *Streptococcus* genus (i.e., *Streptococcus salivarius, Streptococcus parasanguinis, Streptococcus anginosus*) in individuals with IMT/SCA. Furthermore, an increase in the *Bacteroides* genus (i.e., *B. uniformis* and *B. thetaiotaomicron*) was observed in the metagenomes of subjects without IMT/SCA.
v. In the study by Million et al*.*^66^, an investigation into variations in blood culture was conducted among individuals with and without infective endocarditis based on the relative abundance (read counts) of species. The authors initially assessed species with substantial abundance (more than ten reads) in the negative control and identified 14 phylotypes, including *Cutibacterium acnes, Malassezia globosa, M. pachydermatis,* 7 *Sphingomonas, Brevundimonas* sp. DS20*, Exiguobacterium* sp. S17*, and Treponema denticola*. Subsequently, they examined species found in more than 50% of cases (more than five valves) and identified the presence of *Staphylococcus epidermidis, Enhydrobacter aerosaccus,* three phages of *Pseudomonas,* two *Prevotella,* and two *Streptococcus* species *(including Streptococcus mitis),* as well as *Paenibacillus sophorae*, *Corynebacterium casei, Lawsonellia clevelandensis,* and *Porphyromonas gingivalis*. After excluding all potential contaminants as defined above, no species could be reliably identified in the heart valves. Some oral bacteria were also detected (*Porphyromonas endodontalis, Prevotella denticola*) in patients negative for endocarditis. *Granulicatella elegans*, previously associated with blood culture-negative endocarditis, along with *Treponema maltophilum* and *Lactobacillus fermentum*, were only identified in isolated cases. Moreover, *Moraxella* species exhibited enrichment in the group with diseased individuals.
vi. Similarly, Mitra et al*.*^61^ reported a similar pattern of bacteria in blood samples. Asymptomatic atherosclerotic plaques (controls) exhibited a greater abundance of Porphyromonadaceae, Bacteroidaceae, Micrococcaceae, and Streptococcaceae species compared to symptomatic atherosclerotic plaques. Symptomatic atherosclerotic plaques displayed elevated levels of Helicobacteraceae, Neisseriaceae, sulfur-oxidizing symbionts, and Thiotrichaceae relative to asymptomatic atherosclerotic plaques. Among the most prevalent oral species identified in symptomatic atherosclerotic plaque samples were Lac*tobacillus rhamnosus, Neisseria polysaccharea, Helicobater pylori,* and *Acidovorax* spp. Notably, the abundance of *Lactobacillus rhamnosus* was considerably higher in the control group compared to the diseased group.

## Discussion

Our secondary data analysis focused on taxonomically identifying metagenomic reads extracted from six studies encompassing a total of 458 samples obtained from individuals diagnosed with specific cardiovascular diseases. The majority of biological samples originated from the gut (447), followed by arterial plaque (7) and cultured blood (4). Across these samples, a total of 17,243 microbial species were identified, with 410 species present in the Human Oral Microbiome Database (HOMD) and subsequently categorized as “oral”. Among these species, 221 and 169 were found in cultured blood and plaque samples, respectively, whereas all 410 were detected in at least one gut samples. From this group, 153 species were identified in the oral-associated environment based on the Human Microbiome Project (HMP) dataset, regardless of their presence in other body sites.

*E. coli* emerged as the most prevalent bacterium within the gut microbiome of CVD patients among the oral bacteria designated by HOMD. While typically considered a gut-dwelling bacterium, research has indicated its presence within the oral cavities of patients with systemic diseases^69,70^. Notably, studies have linked *E. coli* to cardiac infarction, with elevated frequencies in heart attack and stroke patients^59,71^. Additionally, research has revealed the ability of *E. coli*’s lipopolysaccharides (LPS) to permeate cardiac tissues, prompting a thrombogenic response^72^. This same bacterium, along with *Dialister invisus*, has been associated with various oral conditions and strengthens the connection between oral health and CVDs^73–76^.

Considering the hypothesised translocation of oral bacteria to extra-oral sites through mechanisms such as swallowing saliva and bacteraemia, it has been observed that many oral bacteria are present in both gut and blood samples. However, the prevalence of bacteria in the blood stream are low as expected, given that the human body generally maintains blood sterility through various protective mechanisms. These include the presence of a robust immune system that actively detects and eliminates bacteria, physical barriers formed by endothelial cells lining blood vessels, rapid clearance of any invading bacteria, and the low-nutrient nature of the blood that is not conducive to bacterial growth^77,78^. The maintenance of blood sterility is vital for overall health, as bacterial proliferation in the bloodstream can lead to severe conditions like sepsis^16,79–81^.

Notably, *Streptococcus salivarius* emerged as a consistent presence across gut, plaque, and blood samples in our analysis. Previous research has associated this bacterium with various cardiac conditions and chronic inflammation of cardiac tissues^82^. Several periodontal pathogens were also identified in CVD patients, reinforcing the positive correlation between periodontal disease and coronary heart disease^83–86^. Mechanistically, it has been demonstrated that specific oral bacteria have the ability to adhere to and infiltrate endothelial cells, triggering endothelial injury and initiating thrombus formation. Other mechanisms include cross-reactions with endothelial cells and penetration into myocardial tissue, contributing to cardiovascular pathology.

We also noted several periodontal pathogens in patients with CVDs, which proves the positive association between periodontal disease and coronary heart disease^75,76,86–88^. This is similar to a study by Lawrence et al.^89^, where they found that genera related to periodontal diseases such as *Porphyromonas*, *Tanerella* and *Prevotella* are prevalent in patients with CVDs, independently of their respective prevalence being lower that the one of Bacteroides species. Pucar et al*.*^83^ also identified the presence of periodontal bacterial DNA in nine out of 15 coronary artery biopsy samples, with *P. gingivalis* in the highest numbers (53.33%), followed by *Aggregatibacter actinomycetemcomitans* (26.67%), *Prevotella intermedia* (33.33%), and *Tannerella forsythia* (13.33%). Furthermore, bacterial DNA of *Streptococcus mutans*, *P. gingivalis* and *Treponema denticola* species were identified in the atherosclerotic lesions around blood vessels in other studies^90,91^. A study by Ohki et al.^92^ on patients with myocardial infarction showed that red complex bacteria (19.7% *Aggregatibacter actinomycetemcomitans*, 3.4% *P gingivalis*, and 2.3% *Treponema denticola*) are more prevalent compared to other periodontal pathogens. Our analysis yielded results similar to those, in which we identified numerous putative periodontal pathogens in the gut, cultured blood, and plaque samples of patients with CVDs (e.g., *Aggregatibacter actinomycetemcomitans*, *P. gingivalis*, *Prevotella intermedia*, *Tannerella forsythia*, *Treponema denticola*, and *Fusobacterium nucleatum*).

The hemagglutinin genes A and B (HagA and HagB) of *P. gingivalis* can adhere to and enter human coronary artery endothelial cells (HCAECs) inducing endothelial injury and initiation of thrombus formation^93^. In addition, the LPS of *Streptococci* species and *P. gingivalis* can cross-react with endothelial cells, resulting in cell injury, platelet adhesion, and aggregation^94,95^. *P. gingivalis* can even penetrate the myocardium and cause cardiac rupture by releasing matrix metalloproteinase (MMP-9)^96–98^. Another proposed mechanism that could link periodontal disease to CVDs, is the involvement of *P. gingivalis* in foam cell formation and cholesterol efflux by regulating the activities of CD36 scavenger receptors in macrophages^99^. Oral bacteria can also modulate nitric oxide synthase activity, leading to increased nitric oxide production^100^. This production leads to vasodilation of the blood vessels, contributing to hypertension^101^.

Another interesting finding from our analysis was that apart from pathogenic bacteria, many oral bacterial species associated with periodontal health were identified in the gut, cultured blood and plaque samples (Table 2). We also identified some species linked to caries and periodontal disease that are yet to be widely researched for any CVDs. These include *Parvimonas micra*, *Treponema vincentii, Solobacterium moorei, Bulleidia extructa,* and *Lachnospiraceae bacterium*. For instance, *Solobacterium moorei,* a potential oral bacteria associated with halitosis (bad breath) was identified in our analysis and this bacterium was previously reported in blood cultures of patients with bacteraemia, heart failure, and infective endocarditis^102^. *Cutibacterium acnes* was identified in the cultured blood samples in our analysis, however, according to authors and previous literature, this bacterium is also a potential contaminant, and hence this indicated error in sampling^103^. However, one should note that *Cutibacterium acnes* has been associated previously with pericarditis, cardiac tamponade, and constrictive pericarditis^104^. Further studies are needed to explore the role of this oral bacteria in CVDs.

The analysis also highlighted the presence of certain oral bacterial species linked to periodontal health, caries, and periodontal disease within gut, blood, and plaque samples. This emphasizes the underexplored potential of these bacteria in the context of CVDs. Nevertheless, the analysis is constrained by the limited number of studies employing metagenomic sequencing in CVD patients. Factors like heterogeneity in clinical and methodological data, as well as participant characteristics, influence the generalisability of the results. Furthermore, medication effects on microbiomes were not factored in, and additional studies are warranted to better understand oral bacterial transmission and their role in chronic conditions like CVDs. Additionally, future studies exploring the association of specific periodontal pathogens to each CVD is necessary to confirm the association.

Nevertheless, these findings underscore the importance of oral bacterial translocation in CVD patients and illuminate the intricate interplay between oral and systemic health. This study offers a preliminary exploration, encouraging future research to address existing limitations and delve into the potential of novel pathways connecting oral health and cardiovascular well-being. The recognition of oral bacteria in diverse samples of CVD patients reinforces the critical oral-systemic relationship and highlights the oral cavity's ability to exert influence on distant organ systems, including the cardiovascular system.

## Conclusion

Our study found oral bacteria associated with gum and periodontal diseases in the faeces, arterial plaque, and blood samples of CVDs patients. These bacteria were also present in healthy controls, but their types and amounts differed between the two groups. Notably, oral bacteria related to gum health were also seen in CVDs patients. Some of these oral bacteria have not been linked to CVDs before. Common bacteria included *Escherichia, Prevotella, Bacteroidetes, Lactobacillus, Ruminococcus, Eubacterium,* and *Streptococcus. Prevotella* and *Bacteroidetes* levels were higher in healthy controls. Despite this, emerging evidence hints at these oral bacteria potentially contributing to CVDs development. Further research is needed to understand how they impact CVDs initiation and progression.

The discovery of oral bacteria in CVDs patients’ samples emphasises the vital link between oral and overall health. Our study strengthens the idea that the mouth’s condition can affect distant organs, even the heart. To comprehensively understand how these bacteria change with CVDs severity, future studies could investigate their behaviour in both disease and healthy states.

### Supplementary Information

Supplementary Tables.

## Data Availability

The datasets analysed during the current study are available in the original articles cited.
